# Colon Capsule Endoscopy compared to Conventional Colonoscopy under routine screening conditions

**DOI:** 10.1186/1471-230X-10-66

**Published:** 2010-06-18

**Authors:** Julia B Pilz, Susanne Portmann, Shajan Peter, Christoph Beglinger, Lukas Degen

**Affiliations:** 1 Department of Gastroenterology and Hepatology, University Hospital Basel, Petersgraben 4, 4031 Basel, Switzerland; 2 Department of Gastroenterology and Hepatology, University of Alabama Birmingham, Department of Medicine, 1530 3rd Avenue South, Birmingham Alabama 35294-0007, USA

## Abstract

**Background:**

Colonoscopy (CSPY) for colorectal cancer screening has several limitations. Colon Capsule Endoscopy (PillCam Colon, CCE) was compared to CSPY under routine screening conditions.

**Methods:**

We performed a prospective, single-center pilot study at a University Hospital. Data were obtained from November 2007 until May 2008. Patients underwent CCE on Day 1 and CSPY on Day 2. Outcomes were evaluated regarding sensitivity and specificity of polyp detection rate, with a significance level set at >5 mm.

**Results:**

59 individuals were included in this study, the results were evaluable in 56 patients (males 34, females 22; median age 59). CCE was complete in 36 subjects. Polyp detection rate for significant polyps was 11% on CSPY and 27% on CCE.

6/56 (11%) patients had polyps on CSPY not detected on CCE (miss rate).

Overall sensitivity was 79% (95% confidence interval [CI], 61 to 90), specificity was 54% (95% CI, 35 to 70), positive predictive value (PPV) was 63% and negative predictive value (NPV) was 71%. Adjusted to significance of findings, sensitivity was 50% (95% CI, 19 to 81), specificity was 76% (95% CI, 63 to 86), PPV was 20% and NPV was 93%.

**Conclusion:**

In comparison to the gold standard, the sensitivity of CCE for detection of relevant polyps is low, however, the high NPV supports its role as a possible screening tool.

**Trial Registration:**

NCT00991003.

## Background

The incidence of colorectal cancer [(CRC), standardized to age and world population] is 20-45/100.000 for men und 15-30/100.000 for women[1]. It is increasing with age[2] and the cumulative lifetime risk both for men and for women arises to 6%[3]. Regardless of much improved diagnostics, 50% of patients with CRC die from it. The majority (90%) of CRC develop from benign adenomatous polyps. Current evidence points to the important role of screening colonoscopy with subsequent polypectomy (CSPY), reducing the risk of developing CRC by 76-90%[4-6]. Also, early recognization and removal of carcinomatous lesions is crucial, as 5-year survival rates for DUKES stage A (UICC 0 to 1) are above 90%, whereas overall 5-year-survival rate is 62%[7].

Screening guidelines recommending CSPY every 10 years for asymptomatic patients above 50 years with negative family history have been established in several European countries (eg. France, Italy, Germany) as well as in the United States in order to detect polyps and prevent progression into cancer[4,8,9]. Nevertheless, the average miss rates vary from 13% to 27%[10,11] (adenoma ≤1 cm), and around 5% for carcinoma[10]. Moreover, CSPY is an investigator-dependent and risk-bearing (risk of perforation 0.18%, with polypectomy 0.32%)[12] procedure, which requires adequate bowel preparation in order to be cost-effective[13]. Patient acceptance remains the main limiting factor to wide distribution of screening. Correct compliance for screening was observed in 37.1% of subjects and 62.9% for a single CSPY ever in life (retrospective follow-up data in the US (either on yearly FOBT, 5-yearly sigmoidoscopy or 10-yearly CSPY)[14].

For the reason of limited resources, in Switzerland, to date, CRC screening is not regularly promoted by the health-care system[15,16].

Taking all of the above mentioned into account, Colon capsule endoscopy (CCE; PillCam Colon^®^, Given Imaging Ltd., Yoqneam, Israel) might be a novel method for the screening of large populations.

As a further development to the well established PillCamSB^®^, two wide-angle cameras provide an extended view of lesions.

Two previous feasibility studies have shown its possible value for tertiary care centers in a study setting[17,18] as well as practicability in a private practice[19], however, a recent multicenter study evaluating both detection of polyps as well as cancer detected a limited sensitivity[20]. We report the findings of a single center study comparing the performance of CCE with CSPY for the detection of colorectal polyps and cancer.

## Methods

### Study design

This was a prospective, single center pilot analysis comparing the efficacy of CCE against routine screening colonoscopy at a tertiary care center (University Hospital of Basel, Switzerland). Patient enrolment was from November 5, 2007, to May 7, 2008. All patients provided written informed consent. The study was approved by the Ethics Committee and registered at http://www.clinicaltrials.gov (NCT00991003). The study was partly funded by the Nycomed Fund of the University Hospital Basel, Switzerland and by Given Imaging Ltd., Yoqneam, Israel.The authors designed the study, gathered and analyzed the data; the sponsors did not participate in design or conduct of the study nor did they review or approve the data.

### Patients and Data Collection

Every patient who presented at or was referred to our center was considered. Men and women above the age of 50 years without symptoms (indication for screening) or with lower gastrointestinal signs and symptoms and individuals younger than 50 years with positive family history for CRC, minimum 18 years were included in this study. Exclusion criteria were CRC in the patient's history, cardiac pacemaker, contraindications for sodium phosphate solution (Colophos^®^) and risk factors for capsule retention including surgical intestinal anastomosis, Crohn's Disease, diverticulitis and radiologically suspected bowel obstruction.

General patient characteristics were assembled, including demographics, family history and recent surgery, as well as bowel habits. Patient acceptance was assessed with a 5-item questionnaire upon completion of the second examination (Table S1, additional file 1*)*.

Adverse events were recorded on days 1 and 2 of the study. Additionally, some technical data such as completeness of the examinations (inspection of the whole large intestine from ileocoecal valve to anus), colon transit time, location of the capsule at the time of the first Colophos^® ^booster dose and capsule excretion were recorded.

### Study Definitions and Outcomes

The aim was to evaluate this novel method (CCE) for performance as a screening tool compared to CSPY in asymptomatic patients under routine screening conditions. The proclaimed benefit would be an increase in acceptance of screening for CRC and an augmented detection rate of adenoma and/or carcinoma. Significance was defined as polyp size >5 mm, with the hypothesis that detection rate on CCE corresponds with results of CSPY. The primary endpoint was the number of cancerous lesions and polyps detected on CCE compared to CSPY. Secondary endpoints were completeness of the exam, patient acceptance and adherence to preparation regimen. Subanalyses regarding effect of bowel preparation on polyp detection on CCE and accuracy of detection with respect to histopathology were performed.

### Interventions

Patients underwent CCE on day 1 and CSPY on day 2. The examinations were carried out by different physicians, with blinding of results until both examinations had been completed and until interobserver evaluation was finished. CSPY was performed by one of eight different gastroenterologists and intubation of the terminal ileum was not required. CCE was read by two of two gastroenterologists at our department. Segmental unblinding was not feasible as the study was performed during the routine setting at a University Hospital.

**Figure 1:**
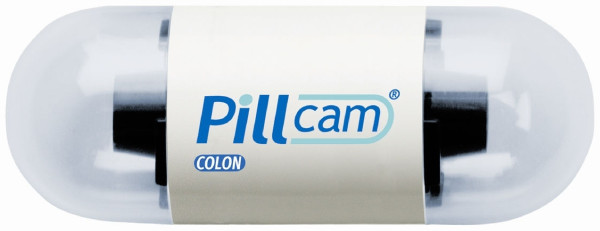
Original size of PillCam^® ^Colon Capsule 11 mm × 31 mm

The PillCam^® ^Colon Capsule is 11 mm × 31 mm in size (Figure 1*)*. It is equipped with two wide-angle (156°) cameras acquiring pictures from both ends of the capsule at a rate of 4 frames per second (2 pictures per second and camera). Activation of the device is automatical on package removal. A sleeping mode of 1 hour 45 minutes is entered upon 10 minutes after ingestion. Detailed procedure has been described elsewhere. Data analysis was performed using RAPID software.

The capsule video was read in three steps: identification of anatomical sites was done during "Quick View Mode" at a rate of 20 frames per second. In a second and third step the pictures were read at approximately 8 frames per second as described before. CCE was read only once in order to simulate a routine clinical setting. Detected lesions were reviewed by an additional physician who assessed their presence, size and localisation, with consecutive interobserver agreement.

Standard CSPY with or without polypectomy was performed under Propofol sedation using existing hospital protocols for preparation, and procedural and post-procedural care[21].

Polyps were estimated by size (greater than 5 mm, ≤5 mm) and location (right or left hemicolon). Polyps detected on CSPY were removed and histologically examined. Other pathologies such as diverticulae, angioectasia and hemorrhoids and their location were also noted but not considered as relevant.

### Colon Preparation, Level of Cleansing and Propulsion of the Capsule

For colon cleansing we applied our department's standard preparation procedure for CSPY including low-fibre diet and Macrogolum (PEG, Cololyt^®^; Spirig Pharma, Egerkingen, Switzerland), and added an oral motility agent (Motilium^®^; Janssen-Cilag AG, Baar, Switzerland), Phospho Soda-boosters (Colophos^®^; Spirig Pharma, Egerkingen, Switzerland) and a suppository (Bisacodylum 10 mg, Prontolax^®^; Streuli Pharma, Uznach, Switzerland), Table 1.

**Table 1:** Summary of bowel preparation

**Time **(hours)	**Action**
	
**Day -3**	
all day	Low-fibre diet
	
**Day -2**	
all day	liquid diet
	
**Day -1**	
all day	clear liquids only
18:00-19:30	2 Liters of Cololyt^® ^(1 cup every 10-15 minutes)
	
**Exam day**	
7:00-8:30	2 Liters of Cololyt^® ^(1 cup every 10-15 minutes)
11:00	20 mg Domperidone (Motilium^®^) with a cup of water
11:15	Ingestion of PillCam^® ^Colon with a cup of water
until 13:00	no food/liquid ingestion
13:00	Real time view assessment if capsule left stomach, then Booster dose of 45 ml Colophos^® ^+ 1 liter of water
17:00	Real time view assessment if capsule left stomach, then Booster dose of 30 ml Colophos^® ^+ 1 liter of water *)
19:30	10 mg Bisacodylum rectal suppository *)
22:00	End of examination
	
**Day +1**	Conventional colonoscopy
	*) This action was taken only if PillCam^® ^Colon had not been excreted by this time.

Quality of colon preparation was assesed using a 3 point scale in both CCE and CSPY, Table 2.

**Table 2:** Description of colon cleansing level

Grade	Description
1	clean, small amount of feces/dark fluid, good assessability
2	moderate preparation due to remaining feces/dark fluid, limited assessability but clean enough to preclude significant lesions
3	poor preparation, enough feces/fluid to strongly limit assessability

### Statistical analysis

CCE was compared to CSPY which was considered to be the gold standard. As this is a pilot study, calculation regarding statistical power did not apply. Sensitivity, specificity, positive predictive value and negative predictive value were calculated per patient, for the included number of patients (n = 56). Percentage values were rounded to the nearest full number. Examinations were excluded from statistical analysis if the capsule had not reached the colon during the recording time. The equivalence between the results of CCE and CSPY was calculated using Wilcoxon signed-rank test and χ2-test. A *p*-value < 0.05 was considered statistically significant. Baseline characteristics, cause of referral, colonoscopic and capsule-endoscopic findings, technical data and results of the patient questionnaire were documented on a datasheet using Excel (Microsoft, Redmont, Washinton, USA). Statistical analyses were performed using SPSS software package (SPSS Inc., Chicago, Illinois, USA).

## Results

### Patients' characteristics

A total of 59 Patients were enrolled in this study. Three patients had to be excluded from the data analysis because the capsule did not reach the colon during examination time (1 remained in stomach, 2 in small bowel).

The exams of 56 patients (34 male, 22 female, mean age 60 years, median 59 years, range 38-84 years, 86% 50 years or older) were analyzed, indications are shown in table 3.

**Table 3:** Indications/symptoms (n = 56)

Screening	23 (41%)
Family history	4 (7%)
Follow-up after polypectomy	3 (5%)
Abdominal pain	8 (14%)
Bleeding frank	8 (14%)
Anemia	2 (4%)
Change in bowel habits	6 (11%)
Diarrhea	1 (2%)
Weight loss	1 (2%)

### Completion of examinations and technical problems

The capsule was excreted within 10 hours after ingestion and within battery duration in 36 patients (64%). In 2 (4%) cases wake-up was too late (past the ileocoecal valve) and in 2 (4%) transmission interference occurred from capsule to data recorder, probably due to external interference (multiple nominations). The mean colon recording time in the 36 complete examinations was 3 hours 9 minutes (median 2 hours 48 minutes). Mean and median transmission times in all 56 subjects were 4 hours 35 minutes and median 4 hours 52 minutes, respectively.

CSPY was complete in all 56 patients, with a rate of 68% (n = 38) for intubation of the terminal ileum.

### Colon Preparation

Level of cleansing on CCE was good in 15 (27%), moderate in 30 (54%) and poor in 11 (20%) cases. On CSPY, 7 (13%) subjects were well prepared, 38 (68%) moderately and 11 (20%) poorly. 34 (61%) of the patients were reported to have the same cleansing level in both colonoscopy and CCE, 22 (39%) had different cleansing levels in both examinations.

### Polyp detection

Polyp detection rate (per-patient) was 50% (n = 28) for CSPY and 62% (n = 35) for CCE. Significant size polyps were diagnosed in 6 patients (11%) on CSPY, resp. 15 patients (27%) on CCE. 11% (n = 6) patients had polyps of any size on CSPY that were not detected on CCE (miss rate) (Wilcoxon signed-rank test *p > 0.05*: not significant).

13/56 (23%) patients had findings of any size on CCE that were not verified on CSPY, 2 (4%) were of significant size. Patients did not undergo repeat colonoscopy.

For polyps of any size, CCE showed a sensitivity of 79% (95% CI, 61 to 90), specificity 54% (95% CI, 35 to 70), PPV of 63% and NPV of 71% for polyps of any size. For overall polyp size, detection of polyps on CCE and on CSPY was independent with statistical significance (*p = 0.013*) on Pearson's *χ*^*2*^-Test, indicating differences in the detection rate for polyps on both examinations (nominal data). For relevant polyps (>5 mm) there was a correspondence in the detection rates of both methods (*p > 0.05*). The sensitivity was 50% (95% CI, 19 to 81), the specificity was 76% (95% CI, 63 to 86), the PPV was 20% and the NPV was 93%, Table 4.

**Table 4:** CCE: Sensitivity, Specificity, PPV and NPV for any size polyps and significant size (>5 mm) polyps

	For polyps of any size	For polyps >5 mm
Sensitivity % (95% CI)	79 (61 - 90)	50 (19 - 81)
Specificity % (95% CI)	54 (35 - 70)	76 (36 - 86)
Positive predictive value %	63	20
Negative predictive value %	71 *Χ*^*2*^*: p = 0.013*	93 *Χ*^*2*^*: p > 0.05*

**Figure 2:**
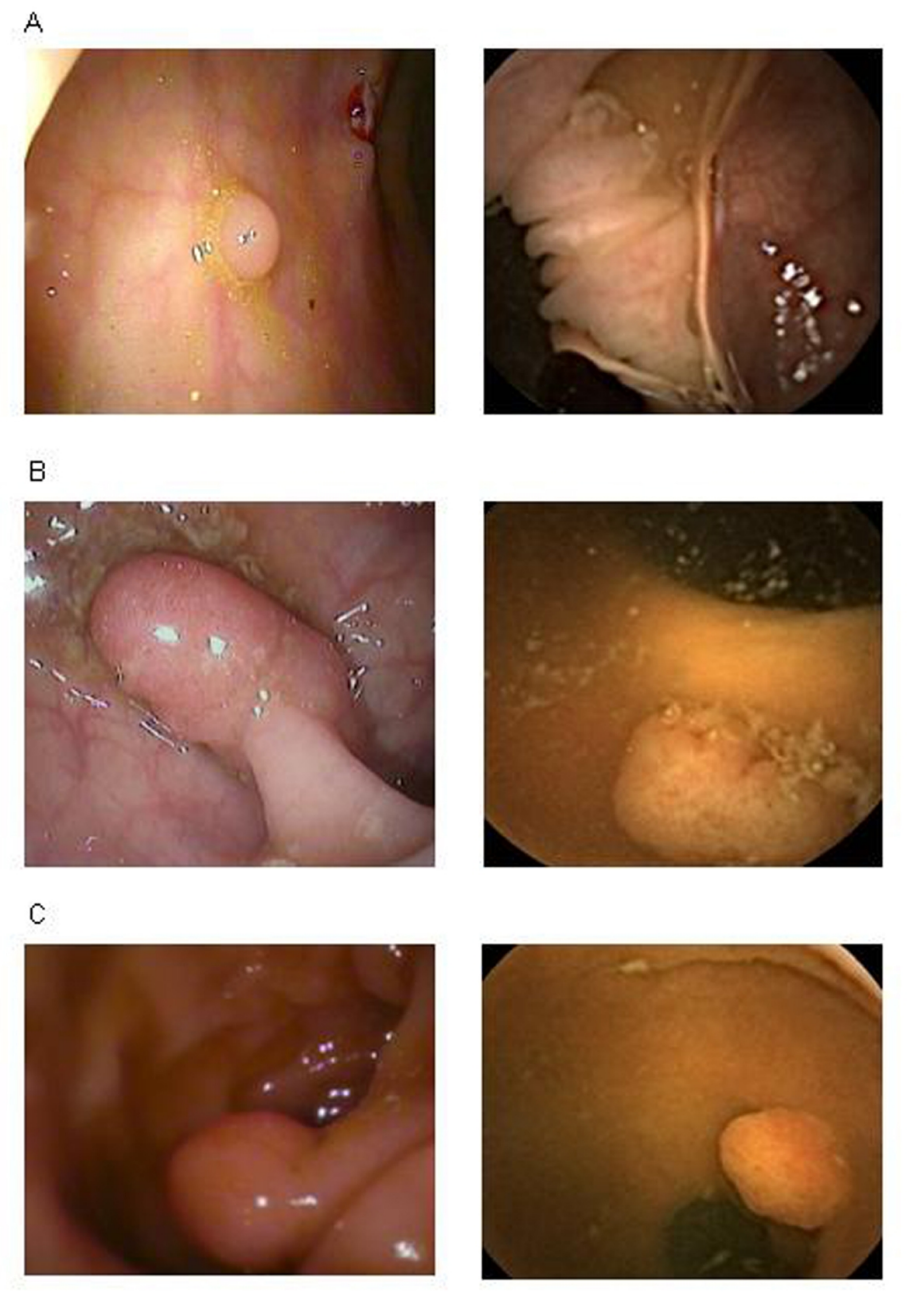
**Images of colon polyps on Colonoscopy (left) vs. CCE (right).** A: Polyp smaller or as big as 5 mm, B, C: Polyps bigger than 5 mm

Presentation of different size-polyps on CCE/CSPY is shown in Figure 2.

### Effect of cleansing level on polyp detection

To assess whether the cleansing level determined accuracy of polyp detection, we subanalyzed the 15 (27%) patients classified as good cleanliness level and the 11 (20%) with a pooply prepared bowel. Sensitivity did not differ significantly in respect to cleansing, although PPV and NPV were both 100% in good bowel preparation.

### Analysis according to histopathology

Subanalysis with respect to histopathology revealed excellent sensitivity for CCE for detection of significant size tubular and tubulo-villous adenoma.

Per-patient-prevalence of adenoma was 27% (n = 15) on CSPY. Numbers are too small to calculate for sensitivity and specificity, but all (n = 3) of the detected tubulo-villous adenoma were detected by CCE. Tubular adenoma were detected in 18% (a total of 10 patients on CSPY, all size), one the two detected tubular adenoma of significant size on CSPY was classified as ≤5 mm on CCE. One of the two detected serrated adenoma was not seen on CCE.No high-grade dysplasia or cancerous lesion was found on either type of examination. Overall prevalence of hyperplastic polyps was 23% (n = 13). A detailed analysis regarding number, size, location and histology of polyps is shown in Table S6 (additional file 2).

### Adverse events

One patient had an allergic skin reaction to the adhesive tape of the electrodes during CCE. One patient presented with abdominal pain after polypectomy (during CSPY). A colonic perforation was ruled out.

### Patients' acceptance

Patients' acceptance was assessed from the given questionnaires. Out of a total of 56 questionnaires, 53 (95%) were returned. No patient reported troubles swallowing the capsule. 16 patients (30%) felt restricted during daily-life activities by carrying the electrodes and the recorder. 21 (40%) patients preferred CCE to CSPY, while 20 (38%) preferred CSPY and 12 (23%) had no preference.

Also, 50 (94%) patients would recommend CCE. 44 (83%) subjects would prefer to undergo a capsule colonoscopy again in ten years' time for screening purposes compared to 8 patients (15%) who declined and 1 patient (2%) who was indecisive.

## Discussion

Overall, this single center pilot trial comparing CCE to CSPY for screening of colorectal cancer showed feasible results in imaging for colonic polyps.

Detection rate of polyps of any size on CSPY was 50%, with an adenoma detection rate of 27% (any size).Those are comparable to a large prospective, community-based study[22].

For the primary endpoint, CCE had a low sensitivity (50%). The used size graduation for significance (as defined as >5 mm in our study and >6 mm/>3 polyps of any size in the previous two pilot studies), is debatable: these definitions are adaptations from studies on virtual colonoscopy (CT and MR colonography) where the significance level was set at >10 mm, showing a sensitivity > 75% and specificity > 90% for both examinations[23-25]. To date, there is evidence that virtual colonoscopy is not an accurate alternative for diagnostically relevant small polyps[26].

Additional polyp detection (of significant size) was 4%. We partly ascribe this to altered size estimation and multiple detection of polyps on CCE[27], and to the generally low detection rate of significant size polyps on colonoscopies in our study (assumption from experience).

However, although numbers are small, CCE showed excellent detection of significant size adenoma (tubuluar and tubulo-villous).

From the 41/56 patients that were diagnosed positive (polyps of any size) on colonoscopy, 6 patients had a negative CCE. Reasons for this miss rate have been discussed above, but clearly, CCE needs improvement to the gold standard.

Several findings on CCE (23%, all polyp size) were not confirmed on CSPY. This could also be a false negative rate for CSPY. As CSPY was referred to as the gold standard, we did not include a second look endoscopy to assess this finding, nor did we analyze underlying patterns why polyps were missed on CSPY.

The negative predictive value of 93% for significant polyps fulfills demands for a screening exam[28], although the meaning is altered by a PPV of 20 to 63%.

A recently published study[20] showed comparable results for sensitivity and negative predictive value, although patients with known history of both polyps and colon carcinoma were included.

Second, the preparation regimen suggested by Given Imaging has much been improved, though it still remains more intense than bowel preparation for colonoscopy.

We found a lower excretion rate for CCE (64%, n = 36) than the two previous pilot studies. This might be caused by an additional fasting time of almost 4 hours between ingestion of the second 2 liters of Macrogolum and initiation of CCE, as motility studies have shown an enhanced colonic propulsion of the capsule through PEG. Also, the Israelian study[18] was able to use Tegaserod (Zelmac^®^, Novartis, Basel, Switzerland) which in Switzerland was discontinued in October 2001.

In our study, we found a good level of cleansing only in 37% (n = 15) patients on CCE. As we regularly use a three-point-scale for graduation of cleansing level this was adapted to CCE for better utility.

We also had a low percentage of good cleansing grades for CSPY (13%, n = 7). Our results for poor bowel preparation are consistent with other reports on colon cleansing, where the mean percentage of poorly cleaned bowels varies between 20-25% (20%, n = 11, in our study)[30,31], but in marked contrast to previous pilot studies that showed an excellent/good cleansing level of 88%[17] resp. 84.4%[18].

Grades for cleansing on CSPY are not described in the two mentioned pilot studies. We do explain our findings with the altered assessment scale and, as mentioned above, at least for CCE, with the break in between ingestion of the second 2 liters of PEG and ingestion of the capsule.

Sensitivity for CCE was not improved by good levels-of-cleansing[17,18,20], however, this might be due to a low number of patients in this subanalysis.

Bowel preparation is also crucial for an efficient colonoscopy. Inadequate preparation correlates with higher polyp miss rates and higher costs due to lower completion rates with need for repeated colonoscopy[25,11].The ideal bowel preparation for CCE is yet to be defined, and timing seems to be important. As for now, if preparation for capsule is more complex than for CSPY, with the procedure itself bearing a risk of volume overload especially in predisposed patients, how large is the benefit of CCE?

Third, 8-10 hours of validated capsule battery duration were not sufficient for all patients, especially those with transit disorders. In a recent motility study for patients with severe intestinal motor disorders, using PillCam SB[32], it was shown, that occurrence of less contractile activity, more static periods and also more content retention was statistically significant. Although these findings apply to small bowel observations, it seems probable to translate affected intestinal motility to the colon and rectum, therefore emphasizing the need for a prolonged battery time.

Fourth, CCE has a good acceptance: 94% of patients (n = 50) would recommend CCE as a screening method to somebody who has so far rejected undergoing screening by CSPY, and 83% (n = 44) would adhere to the suggested screening regimen suggesting a 10-year repeat CCE. Interestingly, on postinterventional assessment, 20 patients (38%) preferred CSPY to CCE which is contradictory to preinterventional data[33-35] and might be due to propofol usage for sedation[36].

## Conclusion

In summary, PillCam Colon provides a screening solution which is minimally invasive, safe and does not require sedation. It is very well accepted by patients and recommended to people who have so far denied CRC screening programs. It is an easy to perform examination with an excellent negative predictive value for application in screening purposes under routine conditions. However, diagnostic accuracy for relevant size polyps (i.e. sensitivity) is low. In order to have an assigned part in CRC screening, capsule endoscopy needs to overcome its main limitations, i.e. adaption in preparation and optimizing of technical aspects.

## Competing interests

The authors declare that they have no competing interests.

## Authors' contributions

JBP designed the study and its concept, obtained funding, acquired, analyzed and interpreted the data, performed the statistical analysis and drafted the manuscript. SP acquired, analyzed and interpreted the data and performed the statistical analysis. SP participated in analysis and interpretation of data and administratively and technically supported the study. CB designed the study and its concept, administratively, technically and materially supported the study, supervised it and critically revised the manuscript for important intellectual content. LD administratively, technically and materially supported the study, supervised it and critically revised the manuscript for important intellectual content. All authors read and approved the final manuscript.

## Pre-publication history

The pre-publication history for this paper can be accessed here:

http://www.biomedcentral.com/1471-230X/10/66/prepub

## Supplementary Material

Additional file 1 **Table S1**. 5-Item Questionnaire Click here for file

Additional file 2 **Table S6: A detailed anaylsis regarding number, size, location and histology of polyps**. Click here for file

## References

[B1] Parkin DM Whelan SL Ferlay J Raymand L Young J (eds) International Agency for Research on Cancer. WHO and International Association of cancer registries Cancer incidence in five continents 1997 Lyon: VII. IARC Scientific Publications

[B2] Krebsmortalität in der Schweiz. (Last accessed 10/15/2009 http://asrt.ch/asrt/newstat/in4ch8605.pdf

[B3] Hawk ET Levin B Colorectal cancer prevention J Clin Oncol 2005 23 2 378 91 10.1200/JCO.2005.08.097 15637400

[B4] Winawer SJ Fletcher RH Miller L Godlee F Stolar MH Mulrow CD Woolf SH Glick SN Ganiats TG Bond JH Rosen L Zapka JG Olsen SJ Giardello FM Sisk JE Van Antwerp R Brown-Davis C Mrciniak DA Mayer RJ Colorectal cancer screening - clinical guidelines and rationale Gastroenterology 1997 112 594 642 10.1053/gast.1997.v112.agast970594 9024315

[B5] Winawer SJ Zauber AG O'Brien MJ Ho MT Gottlieb L Sternberg SS Waye JD Bond JH Schapiro M Panish JF Ackroyd F shike M Kurtz RC Hornsby-Lewis L Gerdes H Stewart ET Prevention of colorectal cancer by colonoscopic polypectomy N Engl J Med 1993 329 27 1977 81 10.1056/NEJM199312303292701 8247072

[B6] Thiis-Evensen E Hoff GS Sauar J Langmark F Majak BM Vatn MH Population-based surveillance by colonoscopy: effect on the incidence of colorectal cancer Telemark Polyp Study I. Scand J Gastroenterol 1999 34 4 414 20 10.1080/003655299750026443 10365903

[B7] American Cancer Society available at http://www.cancer.org

[B8] Levin B Lieberman DA McFarland B Andrews KS Brooks D Bond J Dash C Giardello FM Glick S Johnson D Johnson CD Levin TR Pickhardt PJ Rex DK Smith RA Thornson A Winawer SJ Screening and surveillance for the early detection of colorectal cancer and adenomatous polpys, 2008: A joint guideline from the American Cancer Society, the US Multi-Society Task Force on Colorectal Cancer, and the American College of Radiology Gastroenterol 2008 134 5 1570 95 10.1053/j.gastro.2008.02.002 18384785

[B9] Rex DK Johnson DA Anderson JC Schoenfeld PS Burke CA Inadomi JM American College of Gastroenterology. American College of Gastroenterology guidelines for colorectal cancer screening 2009 (corrected) Am J Gastroenterol 2009 104 3 739 50 10.1038/ajg.2009.104 19240699

[B10] Rex DK Cutler CS Lemmel GT Rhamani EY Clark DW Helper DJ Lehman GA Mark DG Colonic miss rates of adenomas determined by back-to-back colonoscopies Gastroenterology 1997 112 1 24 28 10.1016/S0016-5085(97)70214-2 8978338

[B11] Bressler B Paszat LF Vinden C Li C He J Rabeneck L Colonoscopic miss rates for right-sided colon cancer: a population-based analysis Gastroenterology 2004 127 2 452 6 10.1053/j.gastro.2004.05.032 15300577

[B12] Frühmorgen P Rekto- und Koloskopie. In: Blum AL, Siewert JR, Ottenjann R, Lehr L (eds) Aktuelle Gastroenterologische Diagnostik 1985 Berlin: Springer 266 275

[B13] Rex DK Imperiale TF Latinovich DR Bratcher LL Impact of bowel preparation on efficiency and cost of colonoscopy Am J Gastroenterol 2002 97 7 1696 700 10.1111/j.1572-0241.2002.05827.x 12135020

[B14] Peterson NB Murff HJ Ness RM Dittus RS Colorectal cancer screening among men and women in the United States J Womens Health (Larchmt) 2007 16 1 57 65 10.1089/jwh.2006.0131 17324097

[B15] Schoepfer A Marbet UA Colonoscopic findings fo symptomatic patients aged 50 to 80 years suggest that work-up of tumour suspicious symptoms hardly reduces cancer-induced mortality Swiss Med Wkly 2005 135 45-46 679 83 16453208 10.4414/smw.2005.11033

[B16] Oncosuisse http://www.oncosuisse.ch

[B17] Schoofs N Devière J Van Gossum PillCam colon capsule endoscopy compared with colonoscopy for colorectal tumor diagnosis: a prospective pilot study Endoscopy 2006 38 10 971 7 10.1055/s-2006-944835 17058159

[B18] Eliakim R Fireman Z Gralnek IM Yassin K Waterman M Kopelman Y Lachter J Koslowsky B Adler SN Evaluation of the PillCam Colon capsule in the detection of colonic pathology: results of the first multicenter, prospective, comparative study Endoscopy 2006 38 10 963 70 10.1055/s-2006-944832 17058158

[B19] Sieg A Friedrich K Sieg U Is PillCam COLON Capsule Endoscopy ready for colorectal cancer screening? A prospective feasibility study in a Community gastroenterology practice Am J Gastroenterol 2009 104 848 854 10.1038/ajg.2008.163 19240710

[B20] Van Gossum A Munoz-Navas M Fernandez-Urien I Carretero C Gay G Delvaux M Lapalus MG Ponchon T Neuhaus H Philipper M Costamagna G Riccioni ME Spada C Petruzziello L Fraser C Postgate A Fitzpatrick A Hagenmuller F Keuchel M Schoofs N Devière J Capsule endoscopy versus colonoscopy for the detection of polyps and cancer N Engl J Med 2009 361 3 264 70 10.1056/NEJMoa0806347 19605831

[B21] Heuss LT Schnieper P Drewe J Pflimlin E Beglinger C Risk stratification and safe administration of propofol by registered nurses supervised by the gastroenterologist: a prospective observational study of more than 2000 cases Gastrointest Endosc 2003 57 6 664 71 10.1067/mge.2003.191 12709694

[B22] Barclay RL Vicari JJ Doughty AS Johanson JF Greenlaw Colonic withdrawal time and adenoma detection durng screening colonoscopy N Engl J Med 2006 355 24 2533 41 10.1056/NEJMoa055498 17167136

[B23] Hara AK Johnson CD Reed JE Ahlquist DA Nelson H MacCarty RL Harmsen WS Ilstrup DM Detection of colorectal polyps with CT colography: initial assessment of sensitivity and specificity Radiology 1997 205 59 65 9314963 10.1148/radiology.205.1.9314963

[B24] Schoenenberger AW Bauerfeind P Krestin GP Debatin JF Virtual colonoscopy with magnetic resonance imaging: in vitro evaluation of a new concept Gastroenterology 1997 112 1863 70 10.1053/gast.1997.v112.pm9178678 9178678

[B25] Royster AP Fenlon HM Clarke PD Nunes DP Ferrucci JT CT colonoscopy of colorectal neoplasms: Two-dimensional and three-dimensional virtual-reality techniques with colonoscopic correlation Am J Roentgen 1997 169 1237 42 10.2214/ajr.169.5.9353434 9353434

[B26] Rosman AS Korsten MA Meta-analysis comparing CT-colonography, air-contrast barium enema, and colonoscopy Am J med 2007 120 3 203 210 e4 10.1016/j.amjmed.2006.05.061 17349438

[B27] Brinch K Larsson HB Madsen JL A deconvolution technique for processing small intestine transit data Eur J Nucl Med 1999 26 3 272 6 10.1007/s002590050388 10079319

[B28] Rösch T Eickhoff A Fritscher-Ravens A Eliakim R Arber N The new scopes - broadening the colonoscopy marketplace Digestion 2007 76 1 42 50 10.1159/000108393 17947818

[B29] Fireman Z Kopelman Y Fish L Sternberg A Scapa E Mahaina E Effect of oral purgatives on gastric and small bowel transit time in capsule endoscopy Isr Med Assoc J 2004 6 9 521 3 15373307

[B30] Froehlich F Wietlisbach V Gonvers JJ Burnand B Vader JP Impact of colonic cleansing on quality and diagnostic yield of colonoscopy: the European Panel of Appropriateness of Gastrointestinal Endoscopy European Multicenter Study Gastrointest Endosc 2005 61 3 378 84 10.1016/S0016-5107(04)02776-2 15758907

[B31] Ness RM Manam R Hoen H Chalasani N Predictors of inadequate bowel preparation for colonoscopy Am J Gastroenterol 2001 96 6 1797 802 10.1111/j.1572-0241.2001.03874.x 11419832

[B32] Malagelada C De Iorio F Azpiroz F Accarino A Segui S Radeva P Malagelada JR New insight into intestinal motor function via noninvasive endoluminal image analysis Gastroenterology 2008 135 4 1155 62 10.1053/j.gastro.2008.06.084 18691579

[B33] Wee CC McCarthy EP Phillips RS Factors associated with colon cancer screening: the role of patient factors and physician counseling Prev Med 2005 41 1 23 9 10.1016/j.ypmed.2004.11.004 15916989

[B34] Meissner HI Breen N Klabunde CN Vernon SW Patterns of colorectal cancer screening uptake among men and women in the United States Cancer Epidemiol Biomarkers Prev 2006 15 2 389 394 10.1158/1055-9965.EPI-05-0678 16492934

[B35] Tangka FK Molinari NA Chattopadhyay SK Seeff LC Market for colorectal cancer screening by endoscopy in the United States Am J Prev Med 2005 29 1 54 60 10.1016/j.amepre.2005.03.006 15958253

[B36] Lazzaroni M Bianchi Porro G Preparation, premedication and surveillance Endoscopy 2005 37 2 101 9 10.1055/s-2004-826149 15692924

