# Supplementary material for: Colon Capsule Endoscopy compared to Conventional Colonoscopy under routine screening conditions
Source: BMC Gastroenterol. 2010 Jun 18;10:66. doi: 10.1186/1471-230X-10-66 (PMC2905323; doi:10.1186/1471-230X-10-66)
Supplement: Additional file 1 — Table S1. 5-Item Questionnaire [file 1471-230X-10-66-S1.DOC]

## Table S1. 5-Item Questionnaire

| 1. Did ingestion of the capsule and accomplishment of the exam (i.e. CCE) cause any discomfort ? |
| --- |
| - No |
| - Yes (what?) |
| 2. Did you feel restricted during capsule examination (by sensor array and data recorder)? |
| - No |
| - Yes (By what?) |
| 3. Which exam would you prefer (Capsule vs conventional colonoscopy)? |
| - Capsule |
| - Colonoscopy |
| 4. Would you recommend capsule colonoscopy to people who have so far rejected screening colonoscopy? |
| - No |
| - Yes |
| 5. Would you undergo repeat capsule colonoscopy (in case of negative exam) in 10 years for screening purposes? |
| - No |
| - Yes |
