# Supplementary material for: Colon Capsule Endoscopy compared to Conventional Colonoscopy under routine screening conditions
Source: BMC Gastroenterol. 2010 Jun 18;10:66. doi: 10.1186/1471-230X-10-66 (PMC2905323; doi:10.1186/1471-230X-10-66)
Supplement: Additional file 2 — Table S6: A detailed anaylsis regarding number, size, location and histology of polyps. [file 1471-230X-10-66-S2.DOC]

**Table S6:** A detailed anaylsis regarding number, size, location and histology of polyps

|  | **Colonoscopy**  **Findings** | | | | | |  | **Capsule**  **Findings** | | | | | |
| --- | --- | --- | --- | --- | --- | --- | --- | --- | --- | --- | --- | --- | --- |
| **No** | **Polyps**  >5mm  n/ loc | **Polyps**  ≤5mm  n/ loc | **Histology**  **Adenoma**  tub | t-v | serr | **Hyperplastic** | **Cl** | **Polyps**  >5mm  n/ loc | **Polyps**  ≤5mm  n/ loc | **RHF** | **Passage**  minutes | **Completeness** | **Cl** |
|  |  |  |  |  |  |  |  |  |  |  |  |  |  |
| 1 | 0 | 5 RLH | 2 | 0 | 0 | 1 | 3 | 0 | 2 RLH | 1 | 71 | 1 | 3 |
| 2 | 0 | 1 LH | 0 | 0 | 0 | 0 | 2 | 0 | 1 LH | 0 | 488 | 2 | 2 |
| 3 | 0 | 0 | 0 | 0 | 0 | 0 | 2 | 3 RH | 0 | 0 | 208 | 1 | 3 |
| 4 | 0 | 0 | 0 | 0 | 0 | 0 | 2 | 0 | 0 | 0 | 386 | 1 | 1 |
| 5 | 0 | 1 LH | 0 | 0 | 0 | 1 | 2 | 1 LH | 0 | 1 | 215 | 1 | 1 |
| 6 | 0 | 0 | 0 | 0 | 0 | 0 | 1 | 0 | 0 | 0 | 72 | 1 | 1 |
| 7 | 0 | 0 | 0 | 0 | 0 | 0 | 2 | 4 RLH | 0 | 0 | 168 | 1 | 2 |
| 8 | 0 | 1 LH | 1 | 0 | 0 | 0 | 3 | 0 | 0 | 1 | 105 | 1 | 3 |
| 9 | 0 | 1 LH | 0 | 0 | 0 | 0 | 2 | 0 | 0 | 0 | 17 | 1 | 1 |
| 10 | 0 | 0 | 0 | 0 | 0 | 0 | 2 | 5 LH | 0 | 0 | 398 | 2 | 3 |
| 11 | 0 | 0 | 0 | 0 | 0 | 0 | 2 | 0 | 1 LH | 0 | 516 | 2 | 1 |
| 12 | 0 | 0 | 0 | 0 | 0 | 0 | 2 | 0 | 2 LH | 0 | 231 | 1 | 2 |
| 13 | 0 | 1 LH | 1 | 0 | 0 | 0 | 2 | 11 LH | 0 | 1 | 295 | 1 | 1 |
| 14 | 2 LH | 5 LH | 0 | 0 | 0 | 4 | 2 | 0 | 3 RLH | 1 | 168 | 1 | 2 |
| 15 | 0 | 1 LH | 1 | 0 | 0 | 0 | 2 | 0 | 1 LH | 1 | 492 | 2 | 1 |
| 16 | 0 | 2 LH | 0 | 0 | 0 | 1 | 2 | 0 | 0 | 0 | 310 | 1 | 3 |
| 17 | 0 | 0 | 0 | 0 | 0 | 0 | 2 | 3 LH | 0 | 0 | 375 | 1 | 2 |
| 18 | 0 | 0 | 0 | 0 | 0 | 0 | 2 | 0 | 0 | 0 | 41 | 1 | 2 |
| 19 | 0 | 0 | 0 | 0 | 0 | 0 | 2 | 0 | 0 | 0 | 371 | 1 | 2 |
| 20 | 0 | 0 | 0 | 0 | 0 | 0 | 2 | 0 | 0 | 0 | 45 | 2 | 2 |
| 21 | 0 | 0 | 0 | 0 | 0 | 0 | 2 | 0 | 2 LH | 0 | 425 | 1 | 2 |
| 22 | 0 | 1 RH | 1 | 0 | 0 | 0 | 2 | 0 | 3 LH | 1 | 253 | 1 | 2 |
| 23 | 0 | 0 | 0 | 0 | 0 | 0 | 2 | 0 | 0 | 0 | 355 | 1 | 1 |
| 24 | 0 | 0 | 0 | 0 | 0 | 0 | 1 | 0 | 0 | 0 | 295 | 2 | 2 |
| 26 | 0 | 1 LH | 0 | 0 | 0 | 1 | 2 | 0 | 2 LH | 1 | 135 | 1 | 2 |
| 27 | 2 RLH | 0 | 0 | 0 | 0 | 2 | 1 | 0 | 1 LH | 1 | 447 | 2 | 2 |
| 28 | 0 | 0 | 0 | 0 | 0 | 0 | 2 | 0 | 1 RH | 0 | 520 | 2 | 2 |
| 29 | 0 | 1 LH | 0 | 0 | 0 | 0 | 2 | 0 | 1 LH | 0 | 288 | 1 | 2 |
| 30 | 1 LH | 0 | 0 | 1 | 0 | 0 | 2 | 1 LH | 0 | 1 | 55 | 1 | 2 |
| 31 | 0 | 0 | 0 | 0 | 0 | 0 | 2 | 0 | 0 | 0 | 376 | 1 | 2 |
| 32 | 0 | 0 | 0 | 0 | 0 | 0 | 2 | 0 | 0 | 0 | 162 | 1 | 2 |
| 33 | 0 | 3 LH | 0 | 0 | 0 | 2 | 3 | 1 LH | 0 | 1 | 340 | 1 | 3 |
| 34 | 0 | 2 RH | 0 | 0 | 0 | 1 | 2 | 0 | 1 LH | 1 | 71 | 1 | 2 |
| 35 | 0 | 2 LH | 0 | 0 | 0 | 1 | 3 | 0 | 4 RH | 1 | 502 | 2 | 2 |
| 36 | 0 | 0 | 0 | 0 | 0 | 0 | 2 | 0 | 1 LH | 0 | 508 | 2 | 2 |
| 37 | 1 LH | 0 | 0 | 1 | 0 | 0 | 2 | 1 LH | 2 RH | 1 | 30 | 1 | 3 |
| 38 | 0 | 0 | 0 | 0 | 0 | 0 | 1 | 0 | 1 LH | 0 | 193 | 2 | 1 |
| 39 | 0 | 0 | 0 | 0 | 0 | 0 | 3 | 0 | 0 | 0 | 23 | 1 | 3 |
| 40 | 0 | 5 LH | 0 | 0 | 0 | 2 | 2 | 0 | 3 LH | 1 | 87 | 1 | 1 |
| 41 | 0 | 0 | 0 | 0 | 0 | 0 | 2 | 0 | 0 | 0 | 393 | 2 | 3 |
| 42 | 0 | 1 RH | 0 | 0 | 0 | 1 | 3 | 1 LH | 0 | 1 | 521 | 2 | 3 |
| 43 | 0 | 0 | 0 | 0 | 0 | 0 | 1 | 0 | 1 LH | 0 | 56 | 1 | 1 |
| 44 | 0 | 2 RH | 1 | 0 | 0 | 0 | 3 | 3 LH | 0 | 1 | 59 | 1 | 1 |
| 46 | 0 | 6 RLH | 2 | 0 | 0 | 0 | 1 | 1 RH | 1 RH | 1 | 195 | 1 | 1 |
| 47 | 0 | 0 | 0 | 0 | 0 | 0 | 2 | 0 | 1 RH | 0 | 76 | 1 | 3 |
| 48 | 0 | 3 LH | 0 | 0 | 0 | 1 | 1 | 0 | 0 | 0 | 253 | 1 | 1 |
| 49 | 0 | 0 | 0 | 0 | 0 | 0 | 2 | 0 | 0 | 0 | 572 | 2 | 2 |
| 50 | 0 | 3 LH | 0 | 0 | 1 | 0 | 2 | 0 | 0 | 1 | 24 | 1 | 2 |
| 52 | 0 | 0 | 0 | 0 | 0 | 0 | 3 | 0 | 0 | 0 | 445 | 2 | 2 |
| 53 | 0 | 2 RLH | 1 | 0 | 0 | 1 | 3 | 0 | 0 | 1 | 483 | 2 | 2 |
| 54 | 6 RLH | 1 RH | 1 | 0 | 1 | 0 | 2 | 4 RLH | 0 | 1 | 448 | 2 | 2 |
| 55 | 0 | 3 LH | 0 | 1 | 0 | 0 | 2 | 1 RH |  | 1 | 401 | 2 | 2 |
| 56 | 1 RH | 0 | 1 | 0 | 0 | 0 | 3 | 0 | 3 LH | 1 | 438 | 2 | 2 |
| 57 | 0 | 0 | 0 | 0 | 0 | 0 | 3 | 0 | 0 | 0 | 58 | 1 | 1 |
| 58 | 0 | 0 | 0 | 0 | 0 | 0 | 2 | 1 LH | 0 | 0 | 479 | 2 | 2 |
| 59 | 0 | 0 | 0 | 0 | 0 | 0 | 2 | 0 | 0 | 0 | 461 | 1 | 2 |

*Legend: n/ loc=number/ location, with RH=right hemicolon, LH=left hemicolon, RLH=right and left hemicolon, tub=tubular, t-v=tubulo-villous, serr=serrated; RHF=relevant histological findings; Cl=cleansing, with levels 1, 2 and 3 as described before; completeness: 1=complete, 2=incomplete*
